# Early Communicative Development in Williams Syndrome: A Longitudinal Case Study

**DOI:** 10.3390/children10121900

**Published:** 2023-12-08

**Authors:** Eliseo Diez-Itza, Florencia Llona, Verónica Martínez

**Affiliations:** LOGIN Research Group, University of Oviedo, 33003 Oviedo, Spain

**Keywords:** Williams syndrome, early communicative development, joint attention, face preference, pointing, first words

## Abstract

Individuals with Williams Syndrome (WS) have a specific and atypical neuropsychological profile, where language is above what is expected for their mental age, although it shows a late onset. There exists only one longitudinal study in infants younger than 20 months old with WS about early language precursors (joint attention, referential and instrumental behaviors, pointing gesture, verbal tags). The aim of this investigation is to evaluate these precursors in a baby with WS (8 to 18 months). Seven sessions of systematic observation were performed (six at baby’s home, one at the Early Childhood Assistance center). The Battelle Developmental Inventory was used to evaluate the baby’s development in two occasions (12 and 18 months). The results show an atypical development, and he is 5–6 months under what is expected for his chronological age. Attention towards objects prevails over preference for faces, but this one tends to increase. The pointing gesture does not emerge at the end of the observation period and therefore follows the first words that appear. The implications for the comprehension of the early linguistic profile in WS are discussed, as well as the implications for specific intervention strategies in the context of early childhood care.

## 1. Introduction

Williams syndrome (WS) is a neurodevelopmental genetic syndrome caused by hemideletion of 26 to 28 genes in chromosome 7q11.23, and affects 1 in 7500–10,000 people [1]. Its clinical features include congenital heart defects (75%), supravalvular aortic stenosis and peripheral pulmonary artery stenosis. The growth of children with WS is inhibited by infantile hypercalcemia. They present psychomotor retardation affecting balance and coordination. WS is associated with a specific physical phenotype, which includes dysmorphic facial features, with thick and prominent lips, stellate pattern iris, small nose with a sunken bridge, narrow forehead and dental malocclusion, among others [2]. WS was originally described as an infantile cardiological disease, characterized by its own physical phenotype and mild intellectual disability (IQ 50–70) [3,4]. However, very few studies researched behavioral phenotypes, cognitive development, language, and interventions in WS until two decades later [5,6].

### 1.1. Atypical Neuropsychological Profile in WS: Dissociation between Cognition and Language

In 1985, the case of a 14-year-old adolescent girl (Crystal) was referred to Ursula Bellugi, director of the cognitive neuroscience laboratory at the Salk Institute in La Jolla, where the relationship between cognition and language was being studied. Following Crystal’s evaluation, the cases of two other adolescents with WS were investigated and compared with adolescents with Down Syndrome (DS) [7]. In this seminal study, it was concluded that there was a dissociation between language and cognition in WS, as adolescents with WS were functioning at the preoperational stage established by Piaget, and yet possessed language skills that were considered ‘intact’. They could comprehend and produce complex linguistic utterances and had an excellent vocabulary. On the contrary, they presented a significant impairment of visuospatial cognition, manifested in unstructured drawings [7].

Subsequently, Bellugi et al. [8] compared a group of six adolescents with WS and a group with DS of the same age and IQ, which provided specific profiles of both syndromes. It was observed that subjects with WS performed better in verbal tasks and worse in cognitive tasks and, therefore, the profile of language-cognition dissociation with language preservation was confirmed. However, as their research progressed, other European studies showed that their language skills were not completely intact [9,10,11]. These studies point out some phonological, morphosyntactic and lexical alterations and, above all, pragmatic difficulties. These are manifested in the poor coherence and thematic relevance of their conversations, as well as the comprehension of the language, resulting in communication that is less functional than would be expected from their grammatical competence.

At the Salk Institute, an extensive research program of 100 individuals with WS was developed, which made it possible to determine a characteristic profile, defined as an atypical neuropsychological profile, with peaks and valleys. Among the peaks identified were grammar, auditory memory, music, face recognition or social cognition, while in the valleys were pragmatics, visuospatial construction, numeracy, planning or problem solving [12].

In addition, they present a specific personality profile, in which hypersociability, anxiety and empathy stand out [13,14]. It has even been claimed that WS and ASD are at the opposite ends of the sociability continuum [13,15]. However, recent studies have noted similarities between ASD and WS in terms of substantial difficulties in social interaction and pragmatic skills [16,17].

Regarding studies in Spanish, language skills in children, adolescents, and adults with WS have been investigated in the framework of the SYNDROLING project [18], comparing the linguistic profiles of individuals with different neurodevelopmental genetic syndromes at the phonological, morphosyntactic, lexical and pragmatic levels [19,20,21,22,23,24,25,26]; at all levels, these studies have shown specific difficulties and atypical developmental trajectories. Further studies in Spanish have analyzed narrative production [27], lexical-semantic processing [28] and syntax in children and adolescents with WS [29].

### 1.2. Late Onset of Language in Children with Williams Syndrome

Most of these WS studies focused on adolescence, so the profile described does not reflect early development, which presents atypical features that do not correspond to the later profile. This confirms that neurodevelopmental genetic syndromes present an atypical epigenesis with dynamic developmental trajectories [30]. However, there are very few studies on early language development in WS, although existing data show a late onset of language and an atypical pattern of development from the earliest stages [2,31].

In the following, a comparison will be made between the typical early language development in Spanish (TD), according to data from Aguado [32], and the language development in children with WS, according to Garayzábal et al. [2]. A child with TD would begin to produce his first vocalizations between 4 and 5 months, followed by the babbling stage between 5 and 6 months to the age of one year. Only one study of babbling in infants with WS has been conducted, indicating that the reduplicative babbling in Japanese infants with WS appeared with a variable delay from the age of 17 months (68 to 86 weeks) [33]. Once canonical babbling was produced, the onset of first words was recorded from the age of 22 months (88 to 106 weeks). In TD infants, the first words would appear between 9 and 12 months, when they already understand simple commands. In WS infants, the emergence of first words is delayed until after the age of 20 months, due to problems in early phonological processing, whereas at 18–20 months the lexical spurt in TD children has already occurred.

The difference also lies in the fact that children with WS seem to produce their first words even before they understand them well. In fact, their first words appear before understanding or producing deictic pointing gestures, or asking for or giving objects, which are considered referential behaviors of language precursors. For their part, children with TD produce the deictic gesture before speaking, from 9 to 10 months of age. In WS, the transition to complex grammatical structures occurs around four years of age, more than a year later than the children with TD, when they can already inflect verbs and even regulate their play through speech. This grammatical complexity and vocabulary size, although in WS starts with a delay, tends to catch up with the developmental pace of TD children [2,20].

### 1.3. Early Communicative Precursors of Language Development

Several studies have associated preverbal communicative behaviors, such as joint attention, play and imitation, with later language skills [34]. Authors such as Charman et al. [35], Brooks and Meltzoff [36], Thompson [37] and Tomasello [38] argue that the ability to follow gaze and deictic gesture is the basis for “understanding of others”, i.e., social cognition and the later development of theory of mind and, moreover, predicts language development. According to John and Mervis [39], by age 12 months most TD children have entered an intentional stage in which they demonstrate control over their communicative behavior and understanding of the communicative behavior of others. At this age, children can already follow the gaze of another person. These authors maintain that the ability to follow gaze and deictic gesture is an important driver in early communication development, as it serves as a guide in social interactions and referential communication between child and adult. Furthermore, understanding of gaze direction and reaching and grasping movements seems to follow a different trajectory of development than understanding of pointing as a cue to action intention [40].

Longitudinal associations have been demonstrated between joint attention skills (including gaze-following and deictic gesture) and language skills in the second year of life. The emergence of triadic communication is argued to underlie the development of social cognition and representational skills, as well as a way in which reference is established [34]. Mundy and Gomes [41] stated that different aspects of joint attention are differentially related to expressive and receptive language. Morales et al. [42] presented one of the first studies confirming the relationship between joint attention prior to 12 months and expressive vocabulary at age 24 months. Laing et al. [43] showed that initiating joint attention (alternating eye contact between object and person, using deictic gesture, showing) is an important predictor of expressive language, whereas responding to joint attention (following the adult’s gesture) is related to language production and comprehension. These same authors state that the deictic gesture appears around ten months of age and is strongly correlated with language development. Camaioni, Castelli and Longobardi and Volterra [44] showed that the deictic gesture at 12 months predicts later language production. Harris, Barlow-Brown and Chasin [45] stated that the deictic gesture is related to the comprehension of object names. On the other hand, Ungerer and Sigman [46] found that functional play at 13 months was associated with observable language skills 9 months later. Carpenter, Nagell, and Tomasello [47] found that the age of onset of instrumental gesture imitation was moderately related to the age of referential language onset.

### 1.4. Language Precursors in Williams Syndrome

Children with WS show delayed development in the emergence of joint attention skills, not only with respect to chronological age, but also to linguistic abilities [48]. John and Mervis (2010) [39] showed that, despite having higher IQ, preschoolers with WS (mean age 4.14 years) presented greater difficulties in inferring the communicative intent behind deictic gesture and gaze-following compared to children with DS of the same chronological age. Klein-Tasman, Mervis, Lord, and Phillips [49] reported that 34% of their sample of children with WS (mean age 41.59 months) were unable to use adult gaze to locate an object at a distance, even when using the deictic gesture. Lincoln, Searcy, Jones, and Lord [50] found that 20% of their sample (mean 41.6 months) had difficulty following communicative gaze.

In a study by Laing et al. [43] it was observed that although children with WS (17–55 months) had a more expressive vocabulary compared to the DS group, they had difficulty understanding or producing deictic gesture or establishing triadic attention. Another study [51] showed that infants and preschoolers with WS produced an insignificant amount of gaze-following, triadic attention establishment, and fewer distal gestures, including deictic gesture, and fewer conventional gestures. Hepburn, Philofsky, John, and Fidler [52] discuss a dissociation in joint attention in children with WS, stating that the act of initiating joint attention has a delayed onset, whereas the act of responding to joint attention has a developmental similarity to the typical trajectory. This dissociation may alter later social development.

The WS population appears to be ready for word acquisition long before the use of referential gestures. In this sense, Laing et al. [43] and Mervis and Bertrand [53] report that in WS, the production of referential words precedes the deictic gesture. Mervis and Becerra [54] also argue that the late onset of deictic gesture comprehension and production presages significant pragmatic difficulties in infants and preschoolers with WS. In the same vein, Brock (2007) [31] claims that children with WS can acquire vocabulary despite having deficits in joint attention and not yet possessing categorical reasoning skills. With the development of joint attention being a crucial precursor to language acquisition, these authors propose that impairments in these skills may offer important clues as to why language in WS follows an atypical trajectory.

Mervis, Robinson, Rowe, Becerra, and Klein-Tasman [55] studied the onset of deictic gesture and referential language in a longitudinal study of 10 children with WS (aged 4–26 months). They concluded that, except for one case, the rest of the sample produced referential labels prior to comprehension or production of deictic gesture. Bertrand, Mervis, Rice, and Adamson [56] investigated joint attention skills in a girl with WS for one year, from 20 to 32 months, and found that the girl did not establish joint attention until after the lexical spurt. In addition, she showed little interest in objects, did not make requests, and preferred to focus on the faces of those with whom she was interacting.

A similar case study was conducted by Stojanovik and James [57] on a child with WS from age 21 to 31 months, and the development of communicative precursors of language was analyzed. In their case, the results indicated that rather than being a strength, social communication was an area of weakness compared to other aspects of development. In addition, the child showed a clear preference for objects and did not direct his gaze toward the adult. Stojanovik and James [57] state that longitudinal studies with children from early childhood are needed to discover and understand the nature of atypical development. To date, we are aware of only three longitudinal studies of the development of these early communicative precursors of language in WS, and only one of them covers ages below 20 months. Thus, to our knowledge, the present study is the first longitudinal research of early communicative development in a Spanish-speaking infant with WS under 18 months of age.

### 1.5. Objectives and Hypotheses

The general objective of the present research was to explore the early communicative precursors of language development in a longitudinal case study of an infant with WS between 8 and 18 months, by means of a systematic naturalistic observation during six periodic sessions. This general objective is further elaborated into the following specific objectives:

(i) To assess general developmental milestones through the application of the Battelle Developmental Inventory (BDI) at the beginning and at the end of the observation period to determine points of developmental strength and weakness in the areas and subareas assessed by the BDI.
(ii) To observe and analyze the evolution of joint attention, attention to objects, attention to the face, and loss of attention.
(iii) To observe and analyze the evolution of referential behaviors: visual convergence, deictic gesture, verbal comprehension and expression, natural gestures, displaying objects.
(iv) To observe and analyze the evolution of instrumental and cognitive behaviors, such as object permanence, object manipulation strategies, construction tasks and play patterns.

Hypothesis: early communicative precursors of language will have a late onset and atypical pattern of development. The child’s attention will be directed to faces to a greater extent than to objects. Referential behaviors will have a late onset and, in particular, the deictic gesture will have a postverbal onset, responding to the atypical neurodevelopmental profile that characterizes WS. There will be shortcomings in the performance of instrumental and cognitive behaviors. Future extensions of difficulties in communication will be predictable in comparison with the degree of loss functionality, including late onset of language.

## 2. Materials and Methods

### 2.1. Participant

The research is based on a longitudinal case study of an infant with WS who, at baseline, had an age of 0;8.22 and, at the end of the study, an age of 1;6.12. The infant, referred to by the pseudonym Daniel, was diagnosed with WS by the Array CGH comparative genetic hybridization test (aCGH) at four months of age, and presented the physical phenotype characteristic of the syndrome, although with no remarkable medical conditions to date. Since his diagnosis, he attends stimulation, physiotherapy, and speech therapy sessions at the public early intervention services. The baby has a 3-year-old sister and lives with both parents, who have given informed consent for the study, which has been approved by the ethics committee of the University of Oviedo within the framework of the SYNDROLING Project.

### 2.2. Instrument

- Battelle Developmental Inventory (BDI) [58]. This scale allows the assessment of fundamental developmental skills in children from 0 to 95 months. In the present study, the Spanish adaptation of the second edition was applied. The authors state that it is suitable for detecting “at-risk children” at the earliest ages, as well as for identifying developmental strengths and weaknesses, planning treatment, and assessing short- and long-term improvements. The scale consists of 341 items grouped into five developmental areas (a screening mode reduced to 96 items can also be applied). The results are expressed in percentiles, typical scores and equivalent ages by assessment domains and subdomains:
- Adaptative: 59 items evaluating Attention, feeding, dressing, toileting, and personal responsibility.
- Personal/Social: 85 items evaluating Adult interaction, Expression of feelings/affect, Self-Concept, Peer interaction, Collaboration, and Social Role.
- Communicative: 59 items evaluating Receptive Communication and Expressive Communication.
- Motor: 82 items evaluating Muscle control, Body coordination, Walking, Fine motor, and Perceptual motor.
- Cognitive: 56 items evaluating Perception, Memory, Reasoning, Academic skills, and Development of concepts.

### 2.3. Materials for the Naturalistic Observation Sessions

To assess developmental milestones related to joint attention, verbal and nonverbal referentiality, object manipulation, play, and other behaviors that can be observed in interaction situations, a variety of materials were used. These materials were:

- Construction and manipulation toys, such as interlocking pieces to create towers; stackables, such as hoops or cubes; puzzles, dolls, balls, or figures of different characteristics.
- Toys with acoustic sounds, such as rattles, maracas, bells, or tambourines.
- Electronic toys with buttons, digital sound, or movement, such as a screen with farm animals, a microphone, or dolls with voice reproduction or melodies.
- Children’s books made of cloth and cardboard, such as the Pinocchio story or picture books with illustrations of different elements (animals, objects, people, etc.).
- Other objects that responded to the needs of the tasks designed, such as containers (cups, jars), screens made of different materials, ropes, rags, etc.

### 2.4. Procedure

Six observation sessions of the child’s activity in the family home were scheduled together with the parents, at different intervals (1–3 months) throughout the ten months of the study. The sessions lasted approximately one hour and a half and were conducted in the presence of two researchers who presented the observation situations and videotaped them. Each session was planned a week in advance in a meeting that lasted approximately one hour, where the questions, activities and materials needed were prepared. In all sessions, the mother participated in the activities.

- First session (S1): a first contact was made with the baby, his family, and his environment. Daniel’s interaction with objects and adults, his verbal expression, instrumental and object permanence skills, object manipulation and exploration were observed.
- Second session (S2): the materials, activities, and questions necessary for the first application of the corresponding items of the BDI were prepared. In addition, play situations were recorded to register the child’s joint attention. During the activities, simple requests were also formulated to observe the child’s responses.
- Third session (S3): to observe joint attention, an activity was prepared in which two toys were placed in front of the infant, and the adult played with one of them. It was observed whether the child directed and shared attention towards the toy chosen by the adult, ignoring the other one. Object permanence was also evaluated, as it was not fully acquired in the previous session, as well as instrumental behaviors and strategies to reach objects. Comprehension of simple commands, manipulation and attention to objects and referential behaviors were again observed.
- Fourth session (S4): to assess joint attention, an activity was prepared in which it was observed whether the child used communicative references offered by the adult (deictic pointing, gaze direction and verbal instruction) to find an object hidden under some containers. An activity was also designed to measure the child’s attention and anticipation towards an object that, in its trajectory, disappeared behind a screen. In addition, comprehension of simple requests, joint attention in story reading and play situations, verbal expression, and instrumental and manipulative tasks were observed.
- Fifth session (S5): activities were prepared to evaluate progress on: finding a hidden object, anticipating the trajectory of a toy, construction and manipulation tasks, comprehension of simple commands and verbal labels, verbal expression, and play.
- Sixth session (S6): activities, materials and questions were prepared for the second application of the corresponding BDI items. The activities of finding the hidden toy and the trajectory of a toy were carried out, as well as the observation of comprehension of simple instructions, production of verbal labels and joint attention in different play situations.

Table 1, Table 2, Table 3, Table 4, Table 5 and Table 6 show the activities carried out in each session in more detail, describing the number of videos, their total and individual duration, and the content of each one. In addition to the third session, with the appropriate permissions, a 45 min intervention session was recorded in a public center for early care that the child attended weekly (see Table 7).

### 2.5. Data Analysis

The present study is a single-case observational design in which the subject’s behaviors are examined over time. Specifically, it is a diagnostic assessment case study that combines the application of a standardized instrument (BDI) and the systematic observation of selected behaviors of the subject, with the absence of manipulation of independent variables. This type of design is the most suitable one for explaining aspects of a case, indicating in a precise and detailed way the assessment methodology used [59]. Observational methodology in natural or habitual contexts studies the occurrence of perceptible behaviors, so that they can be adequately recorded and quantified. Gardner (2000) [60] explained the methodological issues of naturalistic observation of parent–child interaction.

In order to delimit the wide variety of observational designs, the criteria of the present observational design have been established on the basis of three axes [61]:

- Temporality of the recording (punctual/follow-up)
- Subjects or items observed (idiographic/nomothetic)
- Levels of response (unidimensional/multidimensional).

According to these criteria, the present observational design is a follow-up; idiographic; and multidimensional. Moreover, concurrent data are recorded simultaneously [62], since behaviors can co-occur (e.g., attending, vocalizing, pointing).

The study follows the methods, materials, and design of sessions of classical studies of naturalistic observation of vocalizations and gestures in the transition from prelinguistic communication to speech reviewed by Ninio y Snow [63]. In particular, several studies on gaze-following, pointing, and reaching in infants with typical development [47,64], and with neurodevelopmental disorders [65,66] were considered.

The patterns of appearance, frequency and duration of communicative behaviors that constitute the observational variables of the present study were recorded. The variables were grouped into three categories, for which the corresponding registry tables were constructed:

(i) Attention behaviors:
  - Triadic (joint) attention
  - Attention to objects
  - Attention to face
  - Loss of attention
(ii) Referential behaviors:
  - Picking up, showing and offering objects
  - Visual convergence
  - Deictic pointing: comprehension and production
  - Natural gestures: imitated and spontaneous
  - Responses to requests: verbal and nonverbal
  - Understanding of name and other referents
  - Verbal expression: vocalizations, babbling and verbal labels
(iii) Object permanence and instrumental behaviors
  - Searching for and retrieval of a hidden object
  - Strategies for reaching objects
  - Construction tasks
  - Play: dyadic and object play

These variables were operationalized as follows:

- Duration in time expressed in seconds and proportion of time in percentages (Attention behaviors; Play).
- Frequency of occurrence as a function of time (per minute) (Verbal expression; Natural gestures).
- Frequency of occurrence and proportion as a function of opportunities (other behaviors).

On the other hand, the BDI, both in its first and second administration, made it possible to obtain measures of infant development in percentile and IQ scores, and in age-equivalent scores by domains and subdomains. The results of the BDI and the behavioral observation were presented in Tables and Figures.

## 3. Results

### 3.1. Assessment with the Battelle Development Inventory (BDI)

Based on the overall results of the BDI domains, it was possible to establish a developmental profile for Daniel. The results are shown in Table 8, presented in age equivalents (AE) and correspond to the first (Time 1) and second (Time 2) evaluations, at which moments Daniel had an AE of 1;0.2 and 1;6.12, respectively.

Daniel’s early developmental profile shows Personal/Social (3 months) and Motor (4 months) as the domains of lower development, and Cognitive (7 months) as the domain of higher development, while Adaptive and Communication (5 months) have an AE score that corresponds to the global score. Daniel has made good progress in the BDI areas in the 6-month interval between assessments, as the increase in the global score at the second assessment is 5 months, close to the typical developmental rate. However, the difference between CE and EE, which was already 5 months at the first assessment, increased to 6 months at the second assessment. Therefore, a relative setback of 1 month is observed between the first and second assessment, compared to the typical population. Furthermore, his progress seems atypical, showing an uneven profile with some areas progressing at different rates of development.

To determine the domains of relative strengths and weaknesses and their changing pattern of development from the first to the second evaluation time, the standard deviations in each domain and subdomain with respect to the overall IQ score (Time 1: IQ 86/Time 2: IQ: 72) were used as the criteria. Specifically, a neutral band of one standard deviation (15 points) was considered around the global IQ score, so that half a standard deviation above it (IQ > 93/79) was considered a point of relative strength (+), while half a deviation below it (IQ < 79/65) was considered a point of relative weakness (−). In this case, 65 being the minimum score was always considered a point of weakness. When values were one standard deviation above or below the corresponding overall score, they were considered a special strength (++), or a special weakness (−−). Table 9, Table 10, Table 11, Table 12 and Table 13 present the subdomains of relative strength and weakness at Time 1 and Time 2 based on IQ scores, together with the percentile in the domains and subdomains of the BDI.

The Adaptive area (Table 9), although a point of relative weakness in Time 1, becomes a point of relative strength in Time 2. The same happens with Feeding, being the subdomain with the best evolution. Attention becomes a point of relative strength in the second evaluation.

The Personal/Social domain (Table 10), while remaining in the neutral range, presents a strong asymmetry in the subdomains. Interaction with adults is the best performing and becomes a point of particular strength in the second evaluation; and Expression of feelings and affects maintains a relative strength in the two evaluations. On the other hand, Self-concept is a point of particular weakness, being at the minimum score in both evaluations.

The communicative domain (Table 11) remains in the neutral range in both developmental stages. Receptive communication is maintained as a relative strength in both moments, while Expressive communication exhibits a significant evolution from being a particular weakness in the first evaluation, to falling within the neutral range in the second evaluation.

The Motor domain (Table 12) is the area of the greatest relative regression, becoming a point of relative weakness in the second evaluation. While Body Coordination stands out as a relative strength in the first evaluation, it becomes a point of relative weakness in the second evaluation. The rest of the subareas related to gross motor skills show a regression, becoming points of weakness in the second evaluation. The subareas related to fine motor skills become points of relative (fine motor skills) and special strength (perceptual motor skills).

The Cognitive domain (Table 13) is maintained as an area of special strength in both developmental stages, constituting the area of greatest strength. The evolution of Perception stands out: although it is a point of special weakness in the first evaluation, it becomes a point of special strength in the second evaluation. Memory shows a relative regression, going from being a point of especial strength in the first evaluation to remaining within the neutral range in the second. The remaining sub-areas show a trend of relative evolution, becoming points of relative strength.

### 3.2. Observation of Attention Behaviors

**Figure 1:**
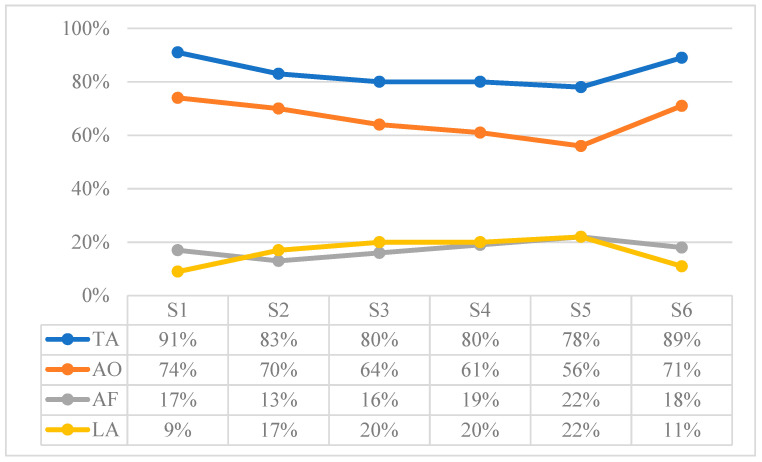
Results in percentage of attention behaviors: Triadic Attention (TA): Attention to Object (AO), Attention to Face (AF); and Loss of Attention (LA).

Table 14 shows the results extracted from the sum of the attention behaviors recorded in each of the sessions, expressed in proportions of duration in seconds. To observe the evolution, Figure 1 shows the results of each session in percentage terms.

The Loss of Attention (LA) in the Triadic Attention (TA) situations increases up to S5, but a significant change in trend is observed in the last session (i.e., the percentage of loss of attention is reduced by 50%). In addition, the Attention to Object (AO) time is clearly higher than the Attention to Face (AF) time.

### 3.3. Observation of Referential Behaviors

**Figure 2:**
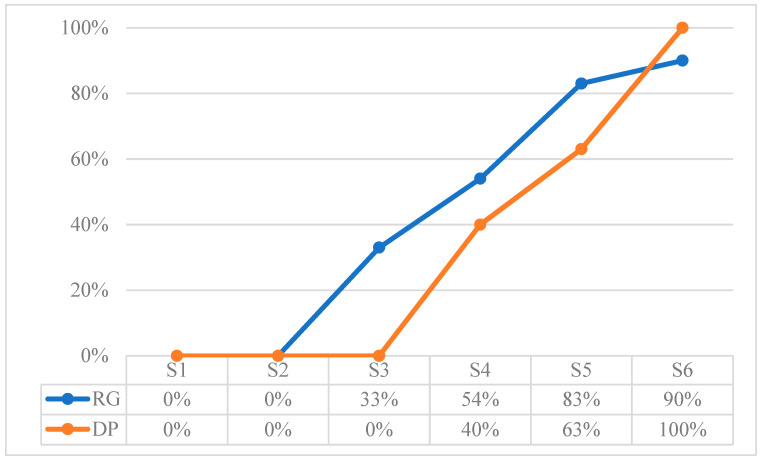
Results in percentage of comprehension of referential behaviors: Referential Gaze (RG), and Deictic Pointing (DP).

**Figure 3:**
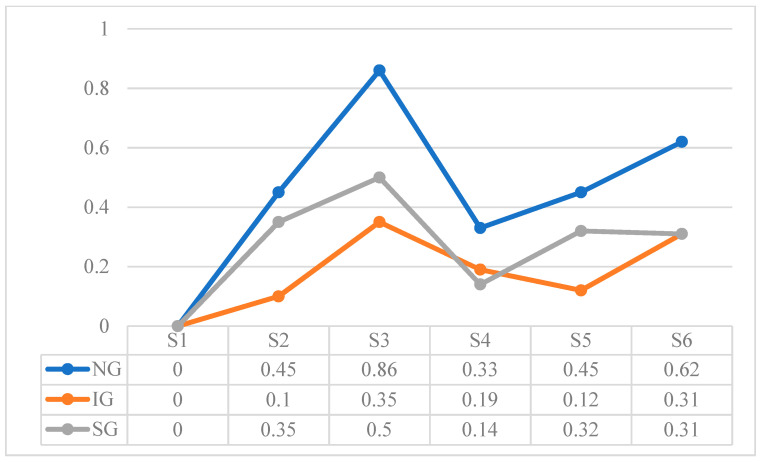
Frequency of production per minute of Natural Gestures (NG): Imitated Gestures (IG), and Spontaneous Gestures (SG).

**Figure 4:**
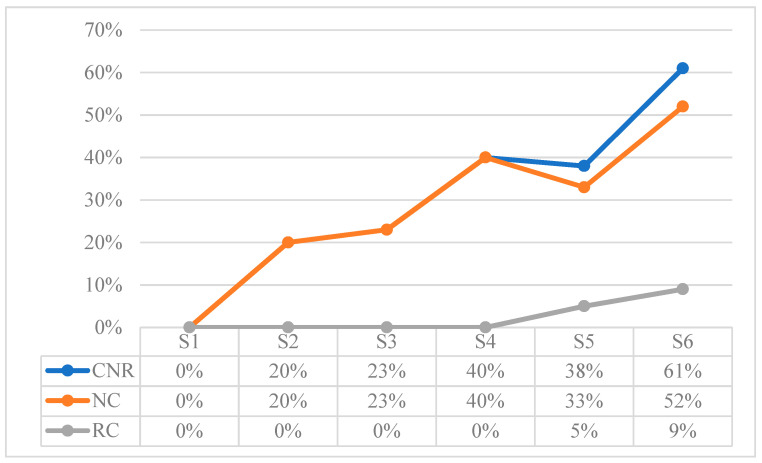
Results in percentage of Comprehension of Name and other Referents (CNR): Name Comprehension (NC), and Referent Comprehension (RC).

**Figure 5:**
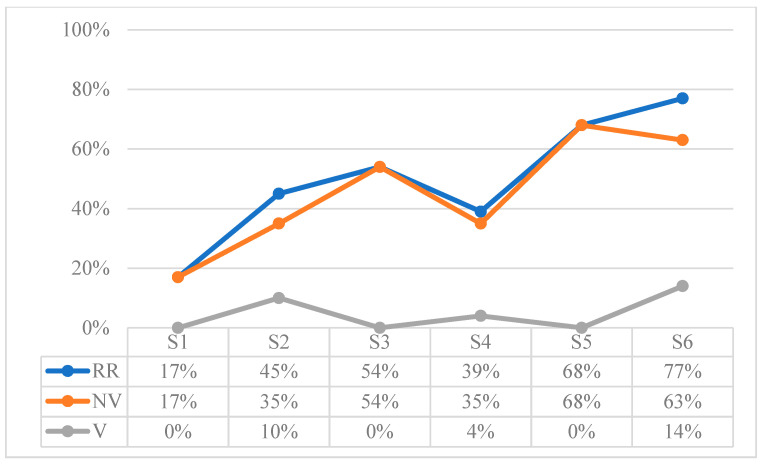
Results in percentage of Response to Requests (RR): requests requiring Non-Verbal response (NV), and requests requiring a Verbal response (V).

**Figure 6:**
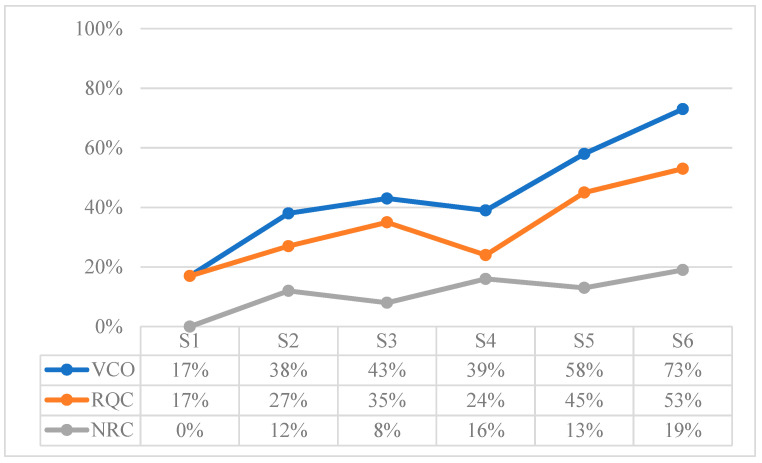
Results in percentage of Verbal Comprehension (VCO): Requests Comprehension (RQC), and Name and Referents Comprehension (NRC).

**Figure 7:**
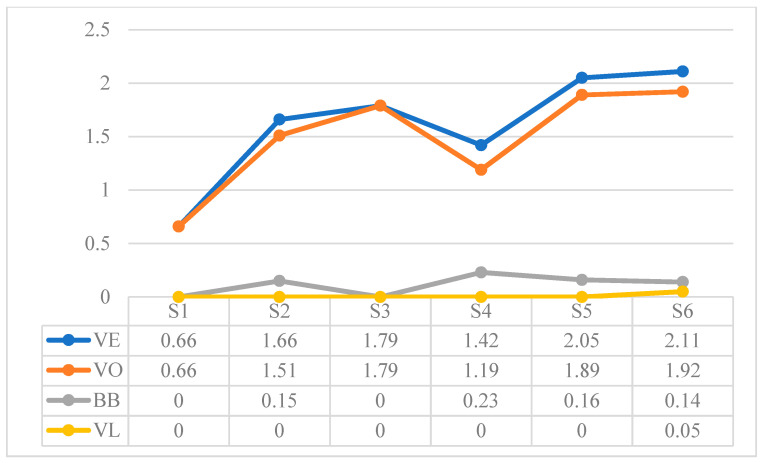
Frequency per minute of Verbal Expression (VE): Vocalizations (VO), Babbling Production (BB), and verbal labels (VL).

Table 15 shows the total values extracted from the sum of the referential behaviors recorded in each session. These results are expressed according to the frequency as a function of time for the Verbal Expression (VE) and Natural Gestures (NG) behaviors, and according to the frequency as a function of opportunities for the rest of the behaviors. To observe the development in more detail, Figure 2, Figure 3, Figure 4, Figure 5, Figure 6 and Figure 7 show the evolution of the different behaviors.

Concerning the behaviors of taking objects (CO) that are offered to him, Daniel takes the objects 100% of the time except in S1. However, it is possible to observe the absence of initiative in terms of showing or offering objects (MO), a behavior that was not observed in any of the sessions.

In Figure 2, the total results of the Referential Gaze (RG) and Deictic Pointing (DP) comprehension behaviors are presented in percentages. The frequency of RG grows from S3 onwards, one step ahead of DP comprehension, which grows from S4 onwards. This pattern of development indicates that the child first understands gaze direction by age 13 months and by age 16 months he also understands the deictic gestures made by an adult to share attention with an object. These behaviors follow with a delay the pattern of typical development, although the production of DP or the use of RG to make requests is not yet present.

Figure 3 shows the frequency of production per minute of natural gestures (NG), differentiating between imitated gestures (IG) and spontaneous gestures (SG). The production of NG follows a changing pattern, with SG occurring more frequently than IG, except for S4 and S6, when the proportion tends to equalize. When the total NG is analyzed, a peak in S3 stands out, with the pattern of appearance having an upward trend in the last three sessions.

Figure 4 shows the results in percentages of the frequency of Comprehension of Name and other Referents (CNR). Name Comprehension (NC) shows an upward trend, while Referent Comprehension (RC) when the referents are named does not appear until S5.

Figure 5 shows the percentages of Response to Requests (RR), which, in general, presents an ascending pattern over the course of the months. However, the reactions to requests entailing non-verbal (NV) type of response appear more frequently than those entailing a verbal (V) type of response. The latter begin to have a greater occurrence at S6.

Figure 6 compares the variables that correspond to verbal comprehension (VCO). It is observed that requests comprehension (RQC) is greater than name and referents comprehension (NRC), i.e., it is greater when requests are made to the child than when the child is called by name or when other referents are named. The VCO has an increasing evolution as the development progresses.

Figure 7 shows the results of the frequency per minute of the Verbal Expression (VE) behaviors. There is an increasing trend of vocalizations (VO), although babbling production (BB) decreases in the last sessions, and verbal labels (VL) occur for the first time in the last session.

### 3.4. Observation of Behaviors Related to Object Permanence and Instrumentation

Table 16 shows the total values of the recording of object permanence and instrumental behaviors in each of the sessions. These results are expressed according to frequency as a function of opportunities for the behaviors of Searching for and retrieving the hidden Object (SO), Strategies for Reaching Objects (RO) and Construction Tasks (CT). Play (PL) is expressed as a function of time in seconds.

**Figure 8:**
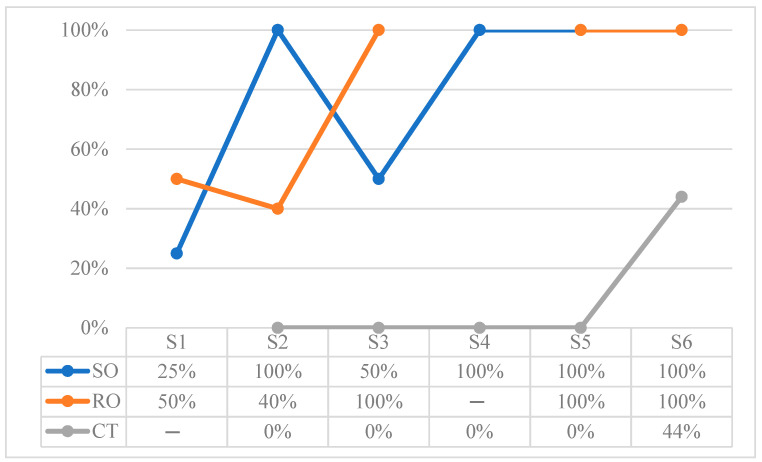
Results in percentage of object permanence and instrumental behaviors: Searching hidden Object (SO), Reaching Objects (RO), and Construction Tasks (CT).

Figure 8 outlines the results in percentages for SO, RO and CT behaviors. Although RO were not evaluated at S4, it can be observed that they are successfully performed from S5 onwards. As for CT, it can be observed that Daniel begins to be successful in its realization from the last session.

Concerning CT behaviors, they were observed only during the last session when Daniel for the first time showed the ability to place figures in the molds and rings in the support, while the construction of a tower with interlocking blocks was not yet performed.

**Figure 9:**
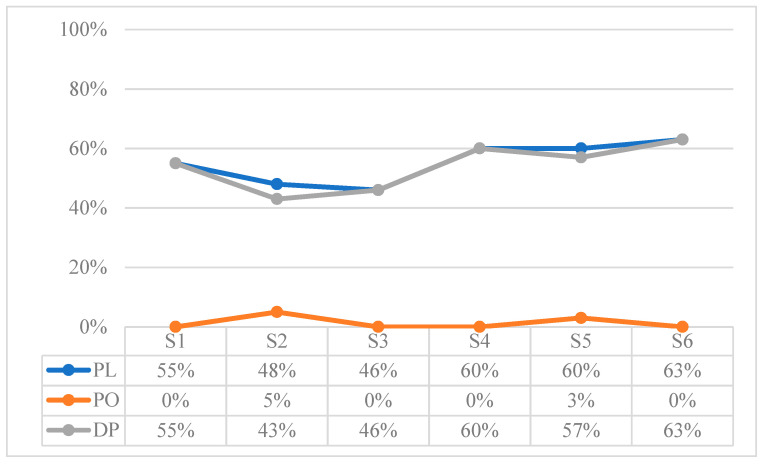
Results in percentage of play behaviors (PL): Play with Objects (PO), and Dyadic Play (DP).

Figure 9 shows the results related to play behaviors (PL) in percentages. Although the duration of PL in each session presents a changing pattern, it is higher in the last three sessions. There is a clear difference between Play with Objects (PO) and Dyadic Play (DP), the latter presenting very low values.

## 4. Discussion

The main objective of the present study was to analyze the early development of communication as a precursor of language in a preverbal infant with WS. As hypothesized, the results confirm a late onset of language and an atypical developmental pattern, with several communicative milestones not having appeared by 18 months. Only three longitudinal investigations of early communicative development in preverbal children with WS have been reported [53,54,55,56,57], only one of which examined infants below 20 months (4 to 26 months).

Although previous studies have highlighted that communicative development in WS equals or exceeds cognitive development [53], this research suggests that communicative skills are an area of relative weakness compared to other areas of development at the youngest ages. The results of the BDI show the contrast between cognitive and communicative behaviors, the former being the greatest strength in this case. This would be in line with the findings of Stojanovik and James (2006) [57] or Brock (2007) [31], who state that younger children with WS do not present the expected communicative skills, not only with respect to the typical population, but also with respect to their general cognitive development.

Regarding the BDI results, it is also worth highlighting those domains of special relative strength in the last assessment of Daniel: Interaction with the adult, an aspect that is consistent with the personality profile that characterizes WS [13]; Perceptual Motor and Fine Motor, and Perception. On the other hand, the subdomains of marked relative weakness are, above all, those related to Gross Motor skills, with the Motor area being the point of greatest weakness in comparison with general development.

Regarding joint attention patterns, contrary to what has been reported in previous research [55,56], the results showed, as in the study by Stojanovik and James [57], that the child had a clear attentional preference for objects, so that attention to the face could not be considered as disruptive to joint attention. This fact could be explained, in this case, by the influence of early stimulation towards objects.

Communicative intention and referential gestures, which may be predicted by the behaviors of showing or offering objects, indicated an absence of initiative in joint attention situations. Since this behavior is a predictor of expressive language [43], such results can be related to the low values observed in verbal expression. The lack of initiative may also be related to the early pragmatic problems that exist in this population. Although the hypersociability of individuals with WS and their facility to engage in conversations is highlighted [2,13]; the development of these social and conversational skills seems to emerge at later stages and presents an atypical pattern. In addition, it has been found that the performance of actions was not accompanied by eye contact, and there is no evidence of the development of adequate joint attention [2,16]. Likewise, although an understanding of referential gaze is observed to occur before the understanding of the deictic gesture, both appear late (15–16 months). Moreover, the production of the deictic pointing did not yet emerge, in line with the studies of Laing et al. [43] and Mervis and Bertrand [53].

As for the production of natural gestures, a peak of improvement was observed in the third session. This is due to an activity that included the percussion of a drum, a toy that is of great motivation and preference for the infant, causing the greatest production of spontaneous gestures.

In relation to verbal expression and comprehension, the results of the BDI and the systematic observation confirmed that verbal expression skills were more affected than verbal comprehension. At 18 months, babbling, production of verbal labels and language comprehension were atypical and delayed for a child of his age [32]. Among the verbal comprehension behaviors, low values of name comprehension stand out. This could be due to Daniel’s perseveration towards objects, which causes an absence of response when the adult tries to capture his attention, rather than to comprehension difficulties.

With respect to behaviors related to object permanence and instructional behaviors, although the results show a late onset, once acquired they maintained a positive pattern of development. These observed behaviors are consistent with some of the items in the Cognitive area of the BDI, which is the greatest strength in this case. The reason could be that these tasks are consistently targeted in the Early Intervention stimulation sessions. An example of this is that Daniel has difficulties with those construction tasks that are not worked on in the Early Care sessions (Towers), but not with others that are stimulated (Hoops and Figures).

The results obtained from the systematic observation of play showed that the child participated mostly in games with objects, in comparison with dyadic play, which hardly appeared. This could be explained by the fact that most of the situations included an object, resulting in very little face-to-face play, which could be considered in future sessions if the longitudinal study were to continue.

It is interesting to highlight the results of two sessions. On the one hand, the values obtained from the recording of the Early Attention session are different when compared with the evolution of the rest of the sessions. The assumption is made that this is due to the long duration of the session, causing Daniel’s tiredness and lack of motivation. On the other hand, the results of the observation of the last session stand to be highlighted, as there is an important change in the tendency of most of the behaviors, with a positive evolution. The explanation could be found in the fact that Daniel, at 18 months, could be starting to show the characteristic change in motivation and interaction that TD children display at 12 months of age.

It is important then to design future research addressing in more depth the specific difficulties encountered in the present study, especially with respect to early referential and communicative behaviors (e.g., production of deictic gestures, gaze-following, making requests). Research on early intervention approaches on these difficulties should be fostered. As stated by Stojanovik and James [57], professionals caring for children with WS should be aware that the cheerful disposition that they show in social situations [13] may mask delayed development and possible limitations in social communication. Therefore, the study of early communication before the onset of language is important for its implications on early intervention, helping to make it more contingent and targeted to the specific needs of children with WS.

We need to acknowledge that the present work shows several limitations, especially those concerning systematic observation as a research methodology at very early ages. Although, in this case, it has allowed a cumulative record of a subject’s growth and development over a period of time, it is important to complement the assessments with standardized tests (in this case, the BDI). In addition, although an attempt was made to create a recording system that collects data as specifically and objectively as possible, it is difficult to achieve total control of a multitude of variables related to the subject and the situation.

## 5. Conclusions

The early communicative development milestones, both in the evaluation through the application of the BDI and in the systematic observation, have appeared late and show an atypical pattern of development. Nevertheless, constant development has been observed in all of the precursors throughout the period studied, with particularly striking improvements in the last session at 18 months.

The pattern of joint attention behaviors shows, in this case, a clear attentional preference towards objects and not so much towards the face as is usually indicated in the literature. Attention to the face tends to increase, which seems to suggest that this is an increasing trend at later ages, perhaps along with interest in language, but that there is no early difficulty in inhibiting a preference for faces.

Referential behaviors show a late onset and atypical developmental pattern. Verbal comprehension is less affected than verbal expression. There is comprehension but no production of referential gestures. Although the first verbal labels begin to appear in the last session, there is still no deictic gesture production, which confirms this strange peculiarity of early development in Williams syndrome.

The development of instrumental and cognitive behaviors shows a late onset. The child succeeds in performing those behaviors that are more frequently promoted in the sessions he attends at the early care center. However, the early care session presents important differences compared to the naturalistic interaction at home and, in general, the interaction is poorer, so the present study could better inform early care in those aspects that are specific to Williams syndrome.

In sum, the results of the present study, the first on a Spanish-speaking infant with Williams syndrome, may shed light on the specific trajectories of preverbal communicative development in individuals with the syndrome. This could provide information to improve predictions about language onset outcomes, and refine early intervention targeting prelinguistic precursors, which have previously been addressed by only a few studies in the literature [2,67]. Specifically, concurrent evidence of postverbal emergence of pointing should be considered for implementing early support for the comprehension and production of referential gestures.

## Figures and Tables

**Table 1:** S1, 22 July (EC = 0;8.22).

	8 Videos (24′6″)	
Video A1: 5′27″	Child–environment interaction. Attention to different toys (train, doll, rattle). Manipulation of different toys (train, doll, rattle).	
Video A2: 1′54″	Object permanence (doll behind opaque screen). Instrumental behavior (doll behind transparent screen).	
Video A3: 1′20″	Object permanence (rattle under a cloth).	
Video A4: 40″	Story reading format.	
Video A5: 5′13″	Joint interaction with different rattles. Dyadic interaction with researcher, “conversation”.	
Video A6: 4′36″	Interaction with cloth book. Manipulation of teether. Playing with toy (shiny necklace), reaching for it.	
Video A7: 2′43″	Dyadic interaction with mother, “conversation”. Vocalizations are encouraged.	
Video A8: 2′10″	Dyadic interaction with researcher, “conversation”. Vocalizations are encouraged.	

**Table 2:** S2, 30 October (EC = 1;0.2).

	10 Videos (19′48″)	
Video B1: 47″	Game of “cuckoo” with the mother and an opaque screen. BDI	
Video B2: 1′45″	Search for the named referent. BDI	
Video B3: 1′1″	Pill manipulation, putting it in and taking it out of a jar. BDI	
Video B4: 52″	Manipulation of a toy screw, putting it in and taking it out of a jar. BDI Dyadic interaction with mother, imitation of vocalizations and gestures. BDI	
Video B5: 5′29″	Pulling hoops in and out of a holder. BDI Exploration and movement. Passing the ball game, mother-baby.	
Video B6: 3′23″	Formulation of simple instructions (“clap your hands, give kisses”).	
Video B7: 2′27″	Instrumental behavior (reaching for doll and rattle behind transparent screen). BDI	
Video B8: 1′41″	Object permanence (rattle and cup). BDI Understanding of “give” and “take”.	
Video B9: 44″	Object permanence (toy and cloth). BDI Manipulation of electronic toy, playing at hitting a wheel	
Video B10: 1′39″	Manipulation of buttons on an electronic toy farm.	

**Table 3:** S3, 23 November (EC = 1;0.25).

	9 Videos (13′54″)	
Video C1: 55″	A ball and a drum are provided, with which the researcher plays. It is observed if the child ignores the ball and pays attention to the drum.	
Video C2: 1′22″	Same activity as in the previous one, with ball. Same activity with ball.	
Video C3: 1′38″	Game of passing the ball in turns, child-researcher.	
Video C4: 1′30″	Moving around the room to reach a toy (ball). Pulling a cloth to reach a toy.	
Video C5: 1′39″	Object permanence (doll and cloth). Understanding “give” and “take”.	
Video C6: 47″	Pulling cloth to reach object. Attention to toy (maraca).	
Video C7: 2′24″	Tracking and searching for a hidden maraca while it sounds.	
Video C8: 1′6″	Use of the index finger to handle electronic clock	
Video C9: 2′34″	Spoon manipulation. Manipulation of comb. Interaction with electronic clock.	

**Table 4:** S4, 19 February (EC = 1;3.22).

	11 Videos (21′)	
Video D1: 1′26″	Removing and inserting hoops from a support.	
Video D2: 51″	Building of a tower with three interlocking blocks.	
Video D3: 1′20″	Building a tower with three interlocking blocks. Unlocking the blocks of the tower.	
Video D4: 17″	Unlocking the blocks of the tower.	
Video D5: 1′24″	Construction of the tower with the mother.	
Video D6: 35″	Story reading format.	
Video D7: 53″	Story reading format.	
Video D8: 5′44′	Cups activity: two plastic cups are arranged and a toy is hidden in one of them. Using a deictic gesture and a look, the child is told where the toy is, so that he/she uncovers the cup.	
Video D9: 1′	Cup activity. Understanding “take” and “give”.	
Video D10: 3′36″	Screen activity: the baby must follow the path of the electronic toy that moves. On the way there is a cardboard screen. It is seen if the child anticipates the place of appearance.	
Video D11: 3′54″	Button manipulation on an electronic toy.	

**Table 5:** S5, 22 March (EC = 1;4.22).

	12 Videos (24′18″)	
Video E1: 1′57″	Understanding of the deictic gesture when pointing to “mama”. Mother–child interaction, with juice. Moving around the room.	
Video E2: 5′23″	Game child–mother–tambourine. Game tambourine–investigator. Understanding “give me”, “take”. Search for hidden tambourine while it sounds. Game of “cuckoo” spontaneous on the part of the child, hides behind tambourine.	
Video E3: 40″	Understanding: “stick out your tongue”, “where is your nose”, “clap your hands”.	
Video E4: 1′36″	Pulling rope to reach tambourine. Triadic interaction, child–investigator–tambourine.	
Video E5: 1′8″	Construction of a tower with cubes with the mother. Construction of tower with the researcher.	
Video E6: 4′35″	Opening a can containing pieces with the mother. Spontaneous play by the baby with the researcher, play drinking from the can. Put pieces through the can slot with the researcher. Spontaneous solitary play with electronic toy table.	
Video E7: 2′18″	Cups activity (understanding referential clues).	
Video E8: 2′7″	Same activity of the cups with small ball.	
Video E9: 1′29″	Activity of guessing the trajectory of the electronic toy.	
Video E10: 19″	Same activity of guessing the trajectory of the toy.	
Video E11: 42″	Same activity of guessing the trajectory of the toy. Spontaneous play with mother, hiding behind the screen.	
Video E12: 2′14″	Attention and movement to reach the microphone that plays sounds. Manipulation of the microphone. Understanding “give” and “take”.	

**Table 6:** S6, 10 May (EC = 1;6.12).

	12 Videos (35′22″)	
Video F1: 5′41″	Child–investigator game—board game.	
Video F2: 3′31″	Game with board with geometric figures.	
Video F3: 2′43″	Game with tambourine, practice of “give” and “take”.	
Video F4: 1′46″	Game with tambourine, understanding simple orders, “give it to mom”.	
Video F5: 2′48″	Story reading format.	
Video F6: 2′13″	Story reading format.	
Video F7: 2′11″	Building and disassembling a tower with three blocks.	
Video F8: 2′25″	Taking out and putting rings on a support.	
Video F9: 1′41″	Game with an electronic turtle that moves and plays music.	
Video F10: 2′16″	Guessing the path of an electronic toy Comprehension: “where are the ears?”, “stick out your tongue”, cow, sheep.	
Video F11: 6′43″	Cup activity: two and three cups with rattle.	
Video F12: 1′25″	Cups activity: three cups and toy screw.	

**Table 7:** Early Care Session, 27 November (EC = 1;1.1).

	1 Video (47′)	
Video SAT: 47′	Reaching for the tambourine by pulling on a string. Pulling hoops in and out of a support. Playing with a small keyboard with sound. Playing with a house, hitting a button. Game with colored cubes containing rattles. Object permanence (rattle and containers). To open a box and to obtain the rattle inside. Stand up with technical support. To open a plastic jar to take out figures. Playing with geometric figures board. To imitate the sound of the Indian Playing with tambourine and sticks, imitating drum. Playing with a baby doll. Playing with bubbles.	

**Table 8:** Scores in the Domains and Subdomains of BDI (Age Equivalent in months).

Domains and Subdomains	Time 1 (12 Months)	Time 2 (18 Months)
Adaptive	7	12
Personal/Social	6	9
Communicative	6	11
Receptive Expressive	8 4–5	15–16 9–11
Motor	6	10
Gross Fine	7 5	9 12
Cognitive	10	17
Global Score	7	12

**Table 9:** Percentile (PC) and IQ in the Adaptive subdomains of BDI.

	Time 1		Time 2	
	PC	CI	PC	CI
Adaptive	6	77 −	9	80 +
Attention	10	81	9	80 +
Feeding	6	77 −	20	87 +
Dressing	-	-	4	74
Personal Responsibility	-	-	-	-
Toileting	-	-	-	-

**Table 10:** Percentile (PC) and IQ in the Personal/Social subdomains of BDI.

	Time 1		Time 2	
	PC	IQ	PC	IQ
Personal/Social	8	79	5	75
Adult Interaction	10	81	37	95 ++
Expression feelings/affect	39	96 +	15	84 +
Self-Concept	1	65 −−	1	65 −−
Peer Interaction	-	-	1	65 −−
Social Role	-	-	-	-

**Table 11:** Percentile (PC) and IQ in the Communicative subdomains of BDI.

	Time 1		Time 2	
	PC	CI	PC	CI
Communicative	17	86	8	79
Receptive	35	94 +	23	89 +
Expressive	1	65 −−	2	69

**Table 12:** Percentile (PC) and IQ in the Motor subdomains of BDI.

	Time 1		Time 2	
	PC	CI	PC	CI
Motor	20	87	1	65 −
Muscle Control	4	74 −	-	-
Body Coordination	38	95 +	1	65 −
Walking	13	83	1	65 −
Gross Motor Score	22	88	1	65 −
Fine Motor Skills	21	88	11	82 +
Perceptual Motor Skills	-	-	24	89 ++
Fine Motor Score	26	90	24	89 ++

**Table 13:** Percentile (PC) and IQ in the Cognitive subdomains of BDI.

	Time 1		Time 2	
	PC	CI	PC	CI
Cognitive	52	101 ++	71	108 ++
Perception	2	69 −−	60	104 ++
Memory	53	101 ++	10	81
Reasoning/Academic Skills	20	87	23	89 +
Development of concepts	-	-	27	91 +

**Table 14:** Total time of attention behaviors (in seconds).

	TA	AO	AF	LA
S1	929/1014	755/1014	174/1014	85/1014
S2	575/694	487/694	88/694	119/694
S3	606/756	488/756	118/756	150/756
S4	982/1226	743/1226	239/1226	244/1226
S5	931/1193	663/1193	268/1193	262/1193
S6	1792/1998	1426/1998	366/1998	206/1998

Note: TA: Triadic Attention, AO: Attention to Objects, AF: Attention to Faces, LA: Loss of Attention.

**Table 15:** Total number of referential behaviors by the number of opportunities and by minute.

	TS/O	GF	DP	CNR	RR	NG	VE
S1	T/O: 9/10 S/O: 0	0/1	0/1	0/5	1/6	--	0.66/m
S2	T/O: 16/16 S/O: 0	0/1	0/1	2/10	9/20	0.45/m	1.66/m
S3	T/O: 14/14 S/O: 0	1/3	0/2	3/13	13/24	0.86/m	1.79/m
SAT	T/O: 19/21 S/O: 0	0	--	8/32	12/21	0.27/m	1.44/m
S4	T/O: 15/15 S/O: 0	7/13	2/5	6/15	9/23	0.33/m	1.42/m
S5	T/O: 9/9 S/O: 0	5/6	5/8	8/21	28/41	0.45/m	2.05/m
S6	T/O: 15/15 S/O: 0	9/10	7/7	14/23	43/56	0.62/m	2.11/m

Note: TS/O: Taking and Showing Objects; GF: Gaze-following DP: Deictic Pointing; CNR: Comprehension of Name and other Referents; RR: Response to Requests; NG: Natural Gestures; VE: Verbal Expression.

**Table 16:** Total number of Object Permanence and instrumental behaviors.

	SO	RO	CT	PL
S1	1/4	1/2	--	791/1446 O: 791/791 D: 0
S2	4/4	2/5	Rings: 0/3 Tower: --	571/1188 O: 511/571 D: 60/571
S3	1/2	4/4	Rings: 0/3	385/834 O: 385/385 D: 0
S4	1/1	--	Rings: 0/3 Tower: 0/3	750/1260 O: 750/750 D: 0
S5	7/7	2/2	Rings: -- Tower: 0/3	892/1458 O: 835/892 D: 57/892
S6	15/15	2/2	Rings: 3/4 Tower: 0/3 Figures: 1/2	1400/2212 O: 1400/2212 D: 0

Note: SO: Searching hidden Object; RO: Reaching Objects; CT: Construction Tasks; PL: Play (O: with objects, D: dyadic).

## Data Availability

The data presented in this study are available on request from the corresponding author. The data are not publicly available due to personal reasons.
